# Performance Evaluation of No-Code Artificial Intelligence Models for the Detection of Acid-Fast Bacilli: A Comparative Analysis of Three Models

**DOI:** 10.7759/cureus.52784

**Published:** 2024-01-23

**Authors:** Yash Arya, Anil R Konduru

**Affiliations:** 1 Pathology, Shri B.M. Patil Medical College, Hospital and Research Centre, Bijapur Lingayat District Educational University, Vijayapura, IND; 2 Pathology, Vels Medical College and Hospital, Vels Institute of Science, Technology and Advanced Studies, Chennai, IND

**Keywords:** real-time training, convolutional neural network, mycobacterium leprae, mycobacterium tuberculosis, acid-fast bacilli, no-code artificial intelligence

## Abstract

Background

Acid-fast bacilli *Mycobacterium tuberculosis* and *Mycobacterium leprae* are the causative organisms behind two major diseases of developing nations, tuberculosis and leprosy, respectively. To efficiently tackle these diseases in developing nations, drugs must be augmented with improved detection modalities. This necessitates the development of enhanced tools that can aid the current detection modalities being used in high-incidence areas. A no-code artificial intelligence model based on image classification is one such tool that can be used in the identification of acid-fast bacilli. This study utilizes three such no-code artificial intelligence models that originate from three different platforms but share identical training, testing, and subsequent evaluation. Thereafter, the study is directed at comparing the three models created and identifying the one that can function as a promising support system for the detection of acid-fast bacilli.

Methods

To begin with, a total of 1000 images per class, i.e., positive and negative for each disease, were captured from the diagnosed slides of tuberculosis and leprosy, taken from the Department of Pathology. Subsequently, these slides were reviewed again by a pathologist to demarcate them as positive or negative for acid-fast bacilli. Once the required number of images was captured, 600 images of each class were selected as the training set, 300 images as the testing set, and the remaining 100 images as the evaluation set. Data augmentation was then performed using techniques such as rotating, mirroring, cropping, and position shifting. These designated data sets were then used to train the image classification software available on the following three platforms: Lobe (Microsoft Corporation, Redmond, Washington, United States), Create ML (Apple Inc., Cupertino, California, United States), Python-based open-source software (PerceptiLabs, Stockholm, Sweden). The final evaluation was based on different parameters such as sensitivity, specificity, ease of use, learning curve, technological resources required, and feasibility of implementation. All parameters put together served the purpose of comparison to identify the most promising model.

Results

Out of the three models tested, the one built using Lobe is the most promising in terms of the evaluation parameters considered. For tuberculosis, the sensitivity and specificity values obtained were 96% each, while for leprosy, they were 100% and 96%, respectively. Also, the model built using Lobe had a near-negligible learning curve, in addition to being the most cost-effective and feasible model to implement. Furthermore, it had a unique real-time training feature, which constantly improved the model throughout the testing period, till the final sensitivity and specificity values were achieved.

Conclusions

In clinical situations where a high number of cases are encountered each day, a no-code artificial intelligence model built using Lobe would get exposed to a huge database, getting trained in real time. Subsequently, such a model would reach considerable levels of sensitivity and specificity and in turn, act as a promising support system for the detection of acid-fast bacilli.

## Introduction

Acid-fast bacilli *Mycobacterium tuberculosis* and *Mycobacterium leprae* are the causative organisms behind two diseases affecting people around the world, tuberculosis and leprosy, respectively. India, along with other developing nations such as Pakistan and Bangladesh, is amongst the top eight countries that contribute to the global tuberculosis burden [1]. As per the India Tuberculosis Report 2022, the total number of incidents of tuberculosis patients (new and relapse) notified during 2021 was 19,33,381, which was 19% higher than that notified during 2020 [2]. A significant dip in notifications occurred during the two major COVID-19 waves. This was due to factors such as changes in the health-seeking behavior of patients and the diversion of human and material resources. However, to achieve the target of eliminating tuberculosis by 2025, the National Tuberculosis Elimination Program in India is adopting strategies such as intensified and active case finding [2]. Such aggressive testing is now necessary to bridge the gap created between the cases found and statistical estimates. Similarly, with India being the biggest contributor to the global leprosy burden, the National Leprosy Eradication Program in India is giving tremendous importance to active case detection and regular surveillance, with the help of the Leprosy Case Detection Campaign (LCDC) [3,4].

This paradigm shift in case detection requirements necessitates the development of enhanced tools that can aid in coping with the increased rates of testing. Conventional laboratory evaluation for the confirmation of these diseases includes smears and histopathological slide evaluation. This is done using routine stains like H&E as well as special stains like Ziehl-Neelsen stain and Fite-Faraco stain. However, interpretation and identification in certain situations, like lower bacillary load and scant samples, make the diagnosis tricky. Specially trained and experienced pathologists and laboratory technicians form an important part of the diagnostic evaluation. Various factors like inexperience and processing-related artifacts, might severely affect the diagnostic process. Combined with the need to comb through hundreds of slides each day, the task in itself becomes herculean and might be prone to fatigue-induced lapses.

This opens up the need for new methodologies and tools to be incorporated into the workflow, which would act as aids to the person screening the slides. One such avenue is the usage of no-code artificial intelligence tools like image classification that can aid in the identification of acid-fast bacilli. Such models can be sequentially trained with pre-identified sets of samples, containing both positive and negative cases. An adequately trained model, placed alongside a laboratory technician working in the field, can boost productivity by aiding in the detection process. Simultaneously, it can contribute towards reducing the chances of an erroneous diagnosis.

Early renditions of such computer-aided diagnosis systems depended heavily on pre-processing, wherein parameters had to be established to account for the morphology of the bacilli, and ranges had to be set. Anything not falling within the pre-set ranges would confuse the system and lead to undesirable results [5]. These limitations were better overcome by the advent of convolutional neural networks, which mimic biological nervous systems. Thus, they attempt to analyze visual imagery, like a human pathologist would. This means that the system learns to identify acid-fast bacilli from scratch, depending more on training, rather than pre-processing [6]. Such a convolutional neural network forms the backbone of any image classification software that after training can become a no-code artificial intelligence model that recognizes acid-fast bacilli.

This study utilized and evaluated three such no-code artificial intelligence models, created on three different platforms, for the detection of acid-fast bacilli. The evaluation was based on different parameters such as sensitivity, specificity, ease of use, learning curve, technological resources required, and feasibility of implementation. All these parameters put together served the purpose of comparison, with the ultimate aim of revealing one model that is most promising as an aid in clinical practice.

## Materials and methods

This study was conducted from August 1, 2022, to September 30, 2022, in the Department of Pathology of Shri B.M. Patil Medical College, Hospital, and Research Centre, Bijapur Lingayat District Educational (BLDE) (Deemed to be University), Vijayapura, India. It was approved by the ethical committee of BLDE (Deemed to be University). For this study, a binocular compound microscope (Olympus CX43, Olympus Corporation, Shinjuku, Tokyo, Japan) with a digital camera attached to it was used. The live digital feed from the camera was transmitted to a computer, running on a Windows operating system (Microsoft Corporation, Redmond, Washington, United States). Apart from this, one computer running on a Mac operating system (Apple Inc., Cupertino, California, United States) and another running on a Linux-based operating system (Linux Foundation, San Francisco, California, United States) were utilized in this study.

**Figure 1:**
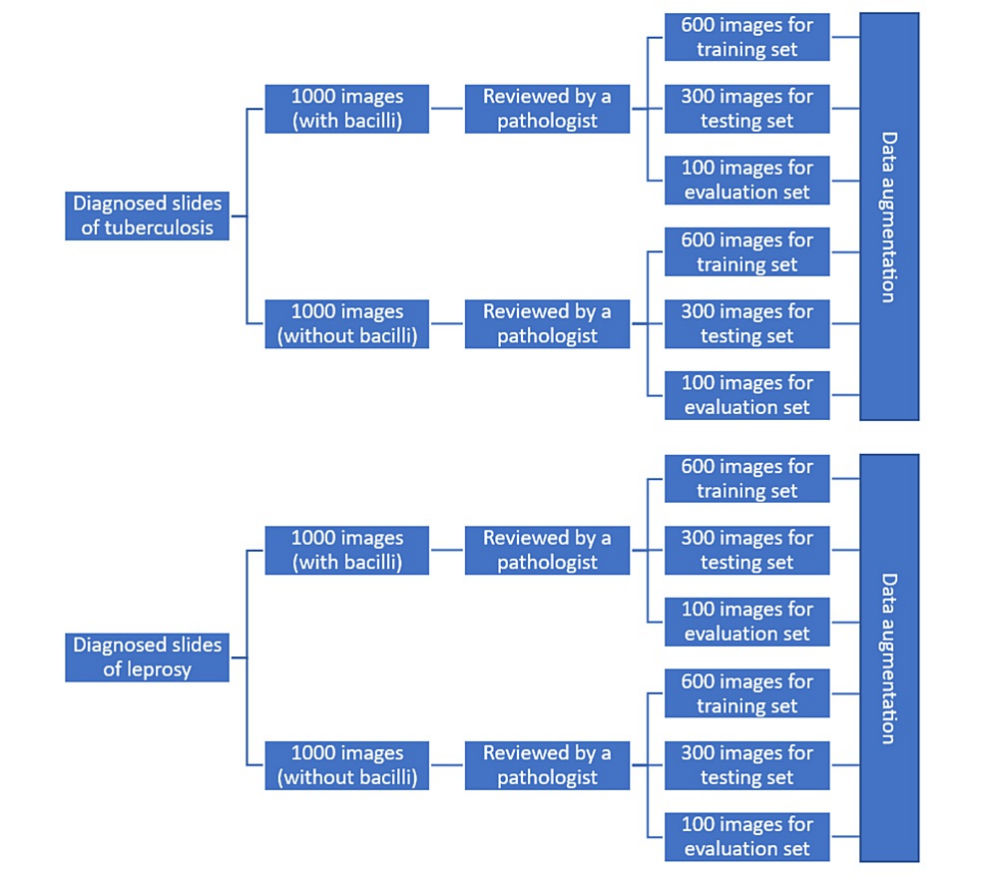
Steps followed for the creation of data sets

A total of 1000 images per class, i.e., positive and negative for each disease, were captured from the diagnosed slides of tuberculosis and leprosy, taken from the Department of Pathology. Subsequently, these slides were reviewed again by a pathologist to demarcate them as positive or negative for acid-fast bacilli. To capture the images, the live digital feed of each slide placed under the microscope was monitored on a computer till the desired field was found. Once that was found, pressing the digital shutter button captured the image and saved it to a predefined folder on the computer. Once the required number of images was captured, 600 images of each class were selected as the training set, 300 images as the testing set, and the remaining 100 images as the evaluation set. Data augmentation was then performed using techniques such as rotating, mirroring, cropping, and position shifting. Figure 1 outlines the various steps followed for the creation of these data sets.

Upon creation, these designated data sets were then used to train the image classification software available on the following three platforms: Lobe (Microsoft Corporation, Redmond, Washington, United States), Create ML (Apple Inc., Cupertino, California, United States), and Python-based open-source software (PerceptiLabs, Stockholm, Sweden). Each of the three models created was first trained using the same training set of images and then tested using the same testing set of images. Subsequently, all three models were evaluated using the same evaluation set of images. Further evaluation of the models was conducted under the following parameters: sensitivity, specificity, ease of use, learning curve, technological resources required, and feasibility of implementation. The various formats used for recording observations under these parameters are listed in Table 1.

**Table 1:** Parameters used for evaluation and the formats used for recording observations under these parameters

Parameters	Formats used for recording observations
Sensitivity	Percentage
Specificity	Percentage
Ease of use	Likert Scale (1 - very difficult to 5 - very easy)
Learning curve	Likert Scale (1 - very difficult to 5 - very easy)
Feasibility of implementation	Likert Scale (1 - very difficult to 5 - very easy)
Technological resources required	Likert Scale (1 - very difficult to execute to 5 - very easy to execute)

## Results

Observations noted after the thorough evaluation of the model created using Lobe are summarized in Table 2. Those obtained after evaluation of the model created using Create ML are summarized in Table 3. Similarly, Table 4 summarizes the observations noted after the evaluation of the model created using Python-based open-source software.

**Table 2:** Summary of the results obtained after the evaluation of the model created using Lobe (Microsoft Corporation, Redmond, Washington, United States)

Parameters	Observations	
	Tuberculosis	Leprosy
Sensitivity	96%	100%
Specificity	96%	96%
Ease of use	5	
Learning curve	5	
Feasibility of implementation	4	
Technological resources required	4	

**Table 3:** Summary of the results obtained after the evaluation of the model created using Create ML (Apple Inc., Cupertino, California, United States)

Parameters	Observations	
	Tuberculosis	Leprosy
Sensitivity	94%	100%
Specificity	100%	93%
Ease of use	4	
Learning curve	3	
Feasibility of implementation	3	
Technological resources required	3	

**Table 4:** Summary of the results obtained after the evaluation of the model created using Python-based open-source software (PerceptiLabs, Stockholm, Sweden)

Parameters	Observations	
	Tuberculosis	Leprosy
Sensitivity	79%	95%
Specificity	90%	82%
Ease of use	2	
Learning curve	1	
Feasibility of implementation	2	
Technological resources required	3	

**Figure 2:**
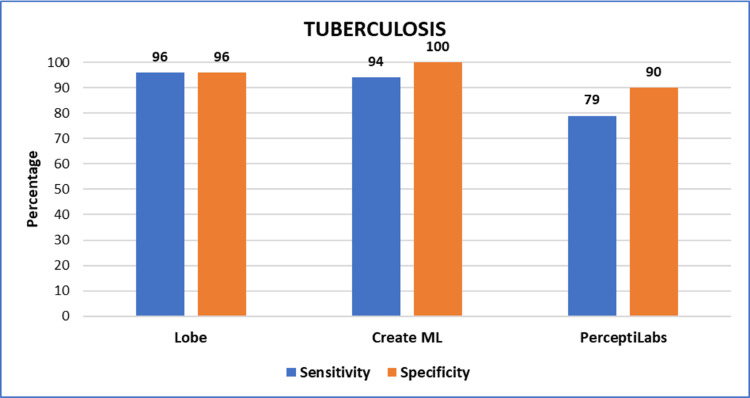
Graph showing the sensitivity and specificity values obtained from all three models for tubercle bacilli detection

**Figure 3:**
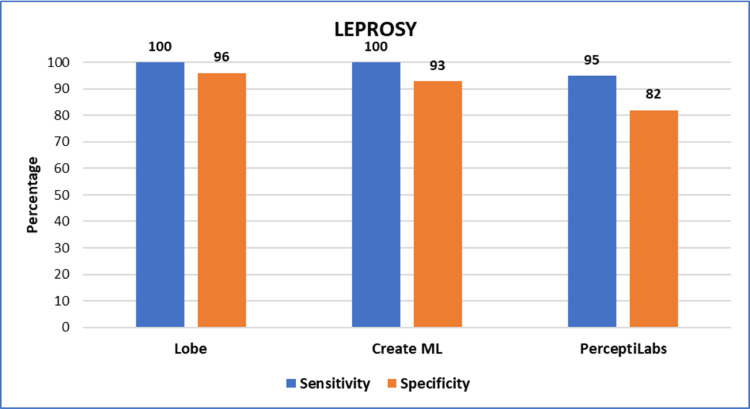
Graph showing the sensitivity and specificity values obtained from all three models for lepra bacilli detection

Figure 2 summarizes the sensitivity and specificity values obtained from all three models for the detection of *Mycobacterium tuberculosis*. Similarly, Figure 3 summarizes the sensitivity and specificity values obtained from all three models for the detection of *Mycobacterium leprae*.

**Figure 4:**
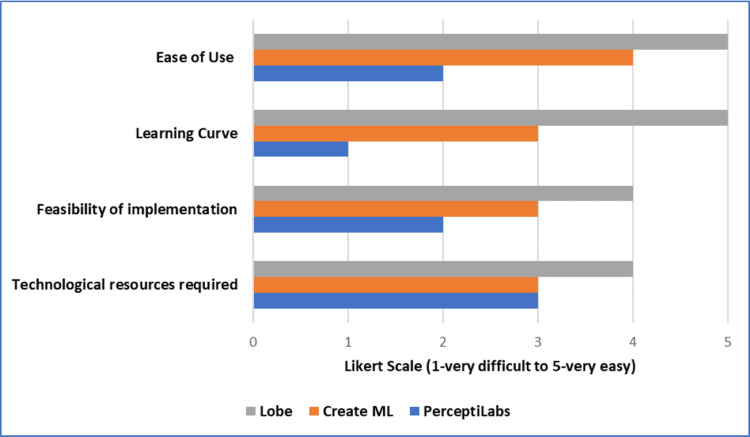
Graph showing the various values obtained from each model for ease of use, learning curve, feasibility of implementation, and technological resources required

Figure 4 summarizes the values obtained from each model for ease of use, learning curve, feasibility of implementation, and technological resources required. Likert scale (1-very difficult to 5-very easy) has been used for the evaluation of these parameters.

## Discussion

Simultaneous training and development of three no-code artificial intelligence models was a process that readily brought out the strengths and drawbacks of each model. During this study, due importance was also given to the key observations made and obstacles faced by authors of previous similar studies, so as to construct an efficient workflow. For instance, del Carpio et al. used whole slide images (WSIs) to train their model [7], while Khutlang et al. used images containing small parts of slides captured by a camera, as their training set [8]. WSIs are much more expensive to create since digital slide scanners are required to capture them; however, their high resolution aids in better bacilli detection. On the other hand, even a relatively cost-effective camera can yield satisfactory results, if paired with a capable convolutional neural network. This was demonstrated by Kuok et al. in 2019 when they used multiple convolutional neural networks to propose a two-stage *Mycobacterium tuberculosis* identification system [9].

**Figure 5:**
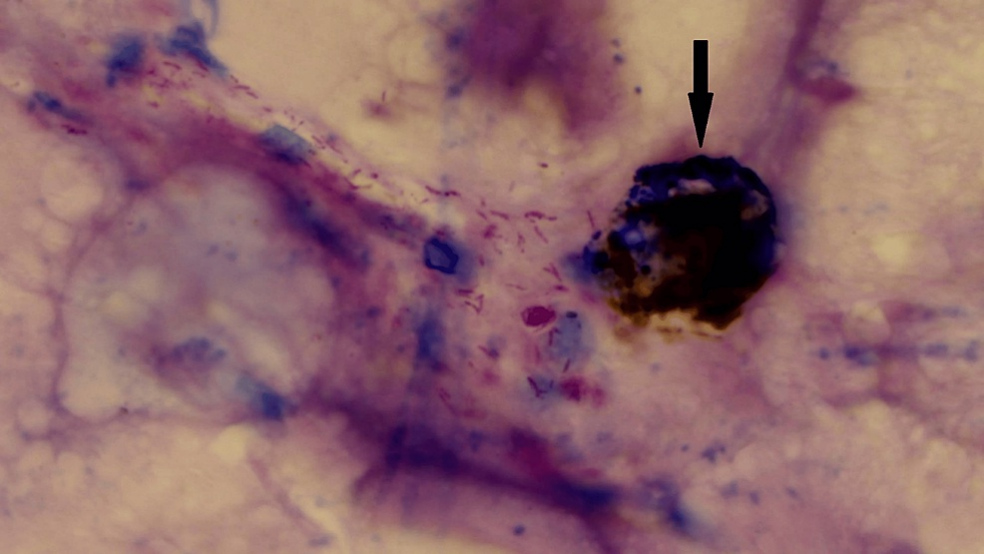
Image of a diagnosed slide of tuberculosis showing an artifact

Another important insight was gained from the contrasting nature of studies conducted by Pantanowitz et al. and Zhai et al. Whole slide images of tissue samples were used in the study conducted by Pantanowitz et al. [10], while the study conducted by Zhai et al. used images of stained sputum smears [11]. From these studies, it was evident that tissue analysis is more difficult than smear analysis and that sectioning of paraffin blocks can lead to the bacilli being cut, along with the generation of more complex artifacts. Thus, the previously conducted studies deemed it possible to create a reliable acid-fast bacilli detection model that was trained using camera-captured images. But before the training of the models even began, proper dataset creation through the collection of slides and their subsequent imaging presented several hurdles. Many of the procured slides showed artifacts under the microscope. The same can be seen in Figure 5.

Some slides did not have uniform staining across the different fields observed. Additionally, the live digital camera feed sometimes struggled with focusing, when a particular field was divided by two or more planes of the same smear. Repeated adjustments to the focus had to be made until the desired segments of the image were visualized clearly. This part of the study was conducted with due patience since Xiong et al. had reported in their study in 2018, that in several cases acid-fast bacilli were found under the microscope, but upon seeing the corresponding digital slides, the bacilli could not be appreciated by the pathologists or their artificial intelligence model [5]. This was due to the limited quality of the digitally scanned images used. They also reported facing the issue of mislabeled data, which necessitated them to run the test data twice. To avoid this, the already diagnosed slides of tuberculosis and leprosy, taken from the Department of Pathology, were again reviewed by a pathologist to demarcate them as positive or negative.

**Figure 6:**
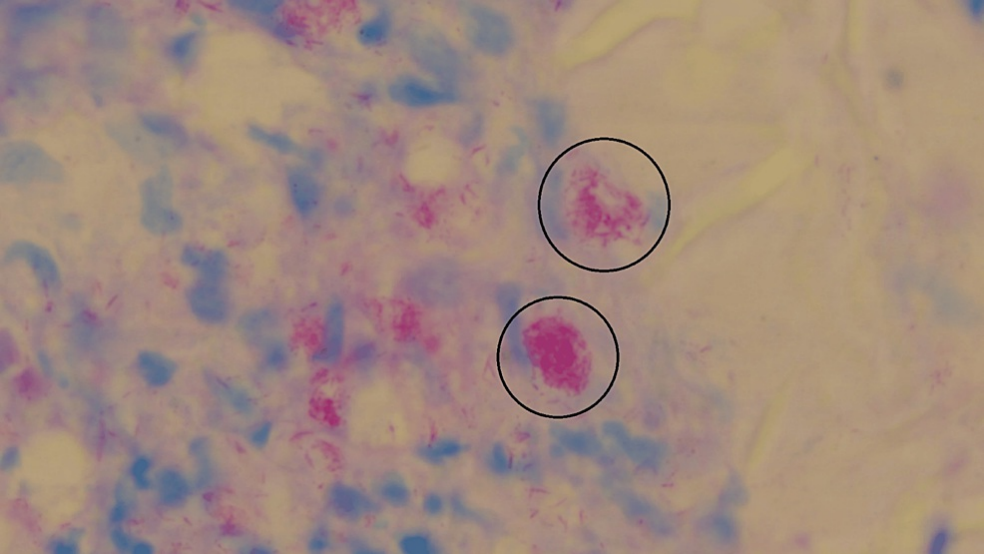
Image of a diagnosed slide of leprosy showing high bacillary load

**Figure 7:**
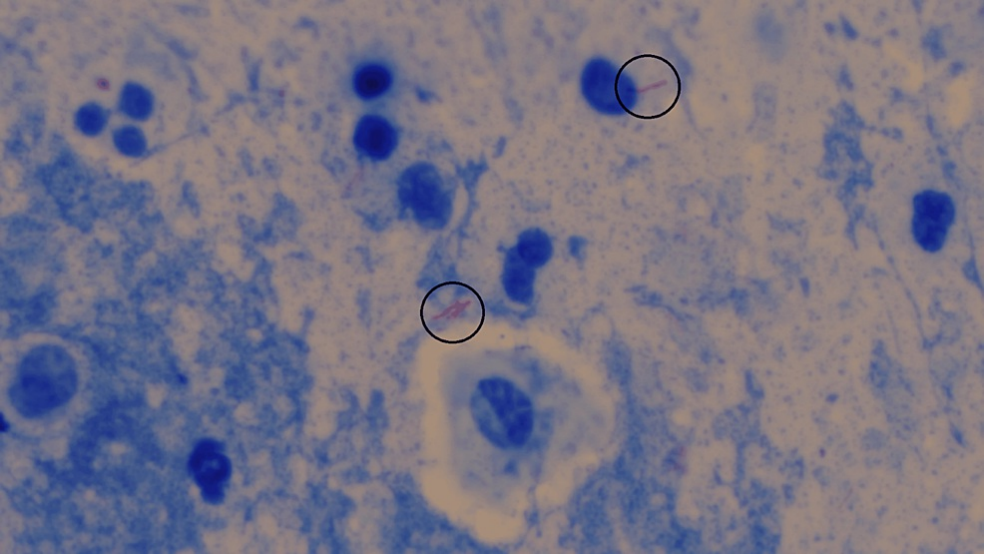
Image of a diagnosed slide of tuberculosis showing low bacillary load

In 2022, the study conducted by Zurac et al. heavily emphasized the need to use a diverse yet balanced dataset while training the artificial intelligence model [12]. To achieve this in the current study, images were captured in such a manner that positive images for each disease comprised an equal number of images with high as well as low bacillary loads. On the other hand, negative images of each disease consisted of some images that had different contrast, brightness, stain particles, etc. Sufficient care was also taken while dividing these images into training, testing, and evaluation sets so that all sets acquired the desired diversity of samples. Such diversity was essential to develop an unbiased model. Figures 6, 7 demonstrate the diversity that was aimed for while creating the datasets.

The first model was created using Lobe. Lobe as a software, consists of a graphical user interface that is very easy to decipher. Upon starting the program, the selection of a structured dataset for training is prompted by the software itself. Training automatically began as soon as the images were uploaded and all the uploaded images could be seen simultaneously. A similar uploading process was repeated for the testing set. Although these steps were completed in a matter of minutes, the drawback of this platform became evident during the evaluation phase. There was no option for uploading all images of the evaluation set together. Hence, individual drag and drop of images was performed, which was a time-consuming process. However, there was an option for real-time training if a response were to be given by the user upon the prediction made by the system.

Overall, the learning curve for this platform was nearly negligible and it was very convenient to use. As such, it can be easily taught to any individual familiar with the Windows operating system, which is amongst the most commonly used operating systems in the world [13]. Also, most of the readily available inexpensive computers can run this software. Hence, the technological resources required are not at all a limiting factor for this model. The sensitivity and specificity values obtained from this model were 96% each for tuberculosis, while for leprosy, they were 100% and 96%, respectively. Thus, the model demonstrated very high values for sensitivity and specificity, despite not being allowed to retrain during evaluation. This indicates the competency of the convolutional neural network at play here.

The second model was created using Create ML. As a software, Create ML was not as intuitive as Lobe, and it took a longer time to decipher the graphical user interface. The selection of a structured dataset for training was similar to Lobe. Unlike Lobe, Create ML had an option for the user to split the training dataset, into training and testing datasets. This feature could be useful in studies with very large datasets, where manual division would be cumbersome. Also, the evaluation dataset could be uploaded simultaneously. Subsequently, the software gave options for changing the number of iterations and adding image augmentation techniques. The training of the model happened at a speed comparable to Lobe, with the results being displayed graphically as well as numerically.

Overall, the learning curve for Create ML was greater than that for Lobe, and it can only be taught to someone already familiar with the Mac operating system, which is not as common as the Windows operating system [13]. Additionally, the cost of purchasing computers that run on the Mac operating system is usually more than the cost of those that run on the Windows operating system [14]. Thus, the technological resources required for this model can become a limiting factor in certain settings. The sensitivity and specificity values obtained from this model were 94% and 100%, respectively for tuberculosis, while for leprosy, they were 100% and 93%, respectively. Thus, this model has the potential to perform well as a confirmatory test for tuberculosis and as a screening test for leprosy. The added benefit of being able to redefine the number of iterations performed can also help in bridging the gap between the values of sensitivity and specificity obtained for each disease.

The third model was created using Python-based open-source software. PerceptiLabs was the least intuitive software used in this study. Developing a working knowledge of Linux-based systems was a mandatory prerequisite to boot the program itself. Once it was booted, familiarization with the interface involved a steep learning curve, the highest among all three platforms. The selection of a structured dataset for training was almost similar to Lobe and Create ML. One key difference was that PerceptiLabs only allowed selecting and uploading one single dataset. Thus, this dataset was divided into training, testing, and evaluation datasets by the software, depending upon the chosen division ratio. An option for data randomization was also present. This feature can greatly boost diversity among the different datasets, in the absence of manual class balancing.

Although a significant drawback emerged in the form of time taken during training, the platform did present a unique option for monitoring the training status. Graphs representing ground truth versus prediction and others showing precision over multiple epochs were visible and constantly updated, throughout the training process. An option to change the code behind the convolutional neural network being used was also available. Thus, PerceptiLabs was the most technically versatile model and gave full control over the algorithm, to the user. Subsequently, the results were displayed both graphically as well as numerically, similar to Create ML. But to implement the use of such a model, computers pre-installed with a Linux operating system need to be acquired and their working knowledge imparted to end users. This would not be very feasible since Linux operating systems are amongst the least prevalent operating systems in the world [13]. Hence, the technological resources required for this model would be a major limiting factor in most setups.

The sensitivity and specificity values obtained from this model were 79% and 90%, respectively for tuberculosis, while for leprosy, they were 95% and 82%, respectively. Thus, despite the versatility offered by this platform, the final sensitivity and specificity values obtained, for either disease, were much lower than the ones obtained from the other two models. The technical complexity offered by the platform could have been a possible deterrent during the creation of this model. While this limitation can be overcome by extensive practice and gathering more information about the workings of a Linux-based system, the same was beyond the scope of this study.

## Conclusions

The ideal no-code artificial intelligence model would be the one that encompasses the strengths of all three models and has the flaws of none of them. A model that is as user-friendly as Lobe, as fast as Create ML, and as versatile as PerceptiLabs, would without doubt perform much better than any of the three models created during this study. Until such an ideal model comes into existence, this study serves the purpose of revealing one model that can be the most useful in clinical practice. Lobe is the clear choice out of the three models in consideration. It is very easy to use, with a near-negligible learning curve, whilst being the most cost-effective and feasible model to implement. Above all, it gives considerably high values of sensitivity and specificity for both diseases. For tuberculosis, the sensitivity and specificity values obtained were 96% each, while for leprosy, they were 100% and 96%, respectively. Furthermore, its real-time training feature has the potential to be very useful in clinical situations where a high number of cases are encountered each day. This would allow the model to be trained indefinitely, amassing a huge database. Thus, this model can indeed be a promising support system for the detection of acid-fast bacilli.
